# Impact of collimator misalignments on dosimetric robustness in proton minibeam radiotherapy using an automated measurement setup

**DOI:** 10.1002/mp.70671

**Published:** 2026-09-07

**Authors:** Sake Salverda, Ernst van der Wal, Thomas Toet, Mischa Hoogeman, Kelvin Ng Wei Siang

**Affiliations:** ^1^ Faculty of Mechanical Engineering Delft University of Technology Delft the Netherlands; ^2^ Department of Research and Development Holland Proton Therapy Center Delft the Netherlands; ^3^ Department of Medical Physics & Informatics Holland Proton Therapy Center Delft the Netherlands; ^4^ Department of Radiotherapy Erasmus MC Cancer Institute Rotterdam the Netherlands

## Abstract

**Background:**

Proton minibeam radiation therapy is an emerging technique that combines the tissue‐sparing benefits of protons with the spatial modulation used for minibeams to reduce toxicity in healthy tissue. Achieving accurate delivery of planned dose distributions depends on precise collimator‐to‐beam alignment, for which it is essential to understand the behavior of both multi‐slit and single‐slit configurations. Understanding single‐slit behavior is particularly important, as it also linearly affects the heterogeneity (peaks and valleys) of the overall minibeam. While several studies have investigated misalignment effects with multi‐slit collimators, there is limited experimental characterisation of single‐slit behavior and its impact on multi‐slit arrays.

**Purpose:**

This study aims to quantify the dosimetric effects of translational and rotational collimator‐to‐beam misalignments for single‐slit proton minibeams using our in‐house automated measurement setup. Additionally, the robustness of lateral dose profiles for minibeam arrays will be investigated under varying collimator misalignments.

**Methods:**

A single slit collimator with 150MeV protons was used to produce a single minibeam with adjustable collimator slit widths (0.4 and 0.7mm). We introduced rotational and translational misalignments using motorized stages. We developed a quick measurement setup to measure lateral profiles using a microDiamond detector. The lateral profiles were measured at depths of 1 and 13cm using a water‐equivalent phantom. We validated the setup by comparing the measurements to gafchromic film measurements using local gamma analysis. A custom fitting model combining two Gaussians and a Lorentzian was developed to fit the lateral profiles. From the fitted profiles, we determined the maximum dose, the lateral position of maximum dose, the full width at half maximum, and the symmetry. Minibeam arrays were simulated via superposition of single‐slit profiles. Specific misalignment combinations were measured for 140, 160, and 170MeV protons, energies typically used in the clinic for deep seated tumours, to assess how misalignments affect the minibeam at different energies.

**Results:**

Rotational misalignments caused substantial asymmetry and peak dose reductions up to 68.4% at shallow depths, with asymmetry reducing at greater depths. Translational shifts resulted in a translation of the minibeam profile in the same direction and magnitude without changing the minibeams shape. Despite these distortions, simulated minibeam arrays achieved acceptable dose homogeneity at 13cm depth for central 80% field‐widths with flatness below a 3% threshold. The proposed measurement setup enabled profile acquisition of a single minibeam in under one minute with submillimetre resolution and strong agreement with gafchromic film (3%/0.2mm gamma pass rates of 100%).

**Conclusions:**

Collimator misalignments substantially affect the minibeam dose robustness and should be considered in clinical treatment planning and quality assurance (QA). The presented measurement setup enables, rapid, high‐resolution profile measurements using commercially available detectors and can facilitate routine clinical QA.

## 1 INTRODUCTION

Proton Minibeam Radiation Therapy (pMBRT) is a novel approach that combines the advantages of spatially fractionated radiation therapy (SFRT) and the tissue sparing properties of protons compared to photons, owing to the characteristic Bragg peak of protons.^1^

With pMBRT, spatially fractionated beams, or minibeams, with a FWHM typically between 0.1mm and 1.0mm,^2^ are used to create a dose pattern of alternating peaks (regions of high dose) and valleys (regions of low dose). The peak‐to‐valley dose ratio (PVDR) quantifies this heterogeneity. With the low dose regions in proximal healthy tissues, pMBRT has shown reduced skin damage and early toxicity in preclinical studies without compromising tumour control,^3^ potentially reducing side effects.^4^ Hypothesised mechanisms include enhanced vascular repair,^5^ regeneration from minimally irradiated cells,^6^ and dose‐volume effects.^6^

Due to multiple Coulomb scattering, proton beams gradually widen with depth, enabling homogeneous dose coverage of the tumour with pMBRT.^6^ However, some studies have proposed spatially modulated (heterogeneous) dose distributions within the tumour which may yield additional immunological benefits.^7^, ^8^

Minibeams can be delivered through static^6^/dynamic^9^ collimation or magnetic focussing.^10^ While magnetic focusing allows higher beam transmission by avoiding beam loss through collimation, collimator‐based methods can be installed on existing clinical systems making these more suitable for widespread implementation. Collimator alignment is a critical issue for pMBRT,^11^ small errors such as a 0.5deg collimator rotation can reduce peak dose and PVDR of the minibeam array by up to 50%. Collimator translations however, result in lateral shifts without altering the lateral profiles shape.^11^ These findings highlight the need to accurately detect and compensate for such errors, especially within a timeframe compatible with clinical workflows. Detecting dose distributions in pMBRT is technically challenging due to its submillimetre resolution requirements.

Previous studies on collimator‐to‐beam misalignments focused mainly on proton minibeam arrays. However, characterising misalignments for single minibeams is crucial so to understand how local variations may average out or compound with a full minibeam array.^12^ This is particularly relevant for PVDR, which is a local dosimetric property sensitive to small‐scale dose variations. Furthermore, a single minibeam serves as a foundational unit of building more complex array structures, linearly affecting their output. Here, we experimentally investigate the effects of translational and rotational misalignments on a single proton minibeam. We developed a detector‐based and system‐independent measurement setup that enables fast measurements of the lateral profile of a single minibeam. In contrast to existing system‐specific approaches, our methodology can be applied to existing clinical and research facilities without requiring detailed knowledge of the beam characteristics or facility‐specific modifications. We then performed an evaluation of how slit width and proton energy influence the resulting lateral dose profiles under collimator‐to‐beam misalignments using this setup. By superposing individual beam profiles, minibeam arrays were simulated to assess how slit‐width, proton energy, and center‐to‐center (CTC) spacing affect the robustness of lateral dose profiles near the entrance and at depth.

## 2 METHODS

### 2.1 Experimental setup

A single planar proton minibeam was generated at the static horizontal research beamline^13^ (Varian Pro‐Beam 4.3) of Holland Proton Therapy Center (HollandPTC) (Delft, the Netherlands) using a 150MeV proton single pencil beam with a central spot, collimated by a 50mm‐thick single‐slit collimator (SSC) composed of two MS58 brass blocks. The SSC is configurable with slit widths of 0.4 and 0.7mm using stainless steel shims (RS Components) placed between the milled surfaces of the brass blocks. The brass blocks were fabricated in‐house via a milling procedure. The 50mm collimator thickness is sufficient to stop protons with energies up to 205MeV.

The collimator was mounted on a stage with two‐degrees of freedom (Thorlabs LTS150/M for translation and Thorlabs HDR50/M for rotation) allowing for *x*‐axis translation and *xz*‐plane rotation, enabling collimator translations and rotations with accuracies <± 2μm and <± 0.05deg, respectively. The center of the collimator was positioned at the iso‐center of the incident beam.

To measure dose profiles at varying depths, water‐equivalent RW3 Slab Phantom, WET 1.04 (PTW‐Freiburg), were used to create the desired water‐equivalent phantom thickness, positioned at an air‐gap of 2.5cm from the collimator.

**FIGURE 1:**
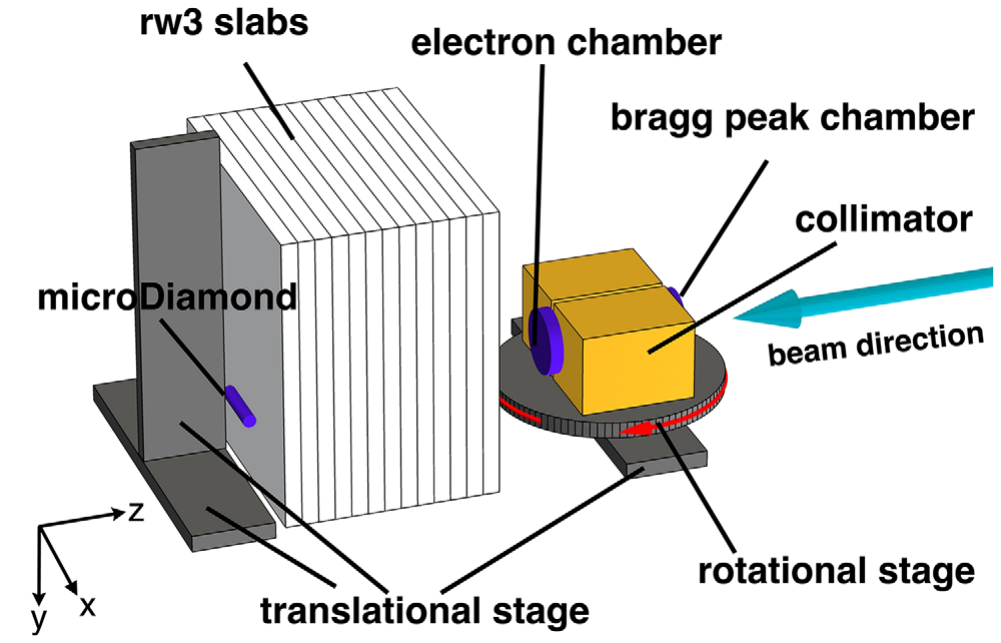
Schematic of the developed experimental setup. The direction of the incoming proton beam is shown in blue. The positive *xz*‐plane rotation is shown in red.

For this setup, the IEC Fixed Coordinate System was used to define the experimental setup, Figure 1. The origin of the *x*‐axis is defined at the central‐axis of the incoming proton beam, and hence the center of the minibeam with nominal alignment.

Table 1 shows the detectors and sensors used, and their usage in the setup.

**TABLE 1:** Overview of used detectors and sensors.

Equipment	Model/manufacturer	Purpose/use	Implementation
**PTW MicroDiamond detector**	PTW‐Freiburg TN60019	Measure minibeam lateral profile.	Mounted on 2‐DOF linear stage (2x Thorlabs LTS150/M), allows translation along x‐ and y‐axis; centerd manually at beam centerline (Y) via laser guides; moved programmatically along X for lateral profile.
**Bragg peak chamber**	PTW‐Freiburg TN34080‐2.5	Measure proton counts; normalize for beam fluctuations.	Placed in front of collimator (beam‐side).
**Advanced Markus electron chamber**	PTW‐Freiburg TN34045	Measure throughput; initially align collimator rotationally and translationally.	Placed on beam central axis behind collimator; removed after initial alignment.
**PTW Unidos Romeo**	PTW‐Freiburg	Read‐out electronics.	Two Unidos used; connected to microDiamond and Bragg Peak Chamber (measurements), connected to Bragg Peak Chamber and Advanced Markus (alignment) to allow simultaneous readouts.
**GafChromic EBT3 film**	GafChromic	Validate microDiamond measurements.	Positioned as required for measurement validation.
**Dial indicator**	Dasqua, accuracy 0.01, range 0‐10mm	Verify rotation stage movement by measuring translational shift of collimator due to rotation.	—

### 2.2 Setup validation

Gafchromic film, the gold‐standard for high‐resolution measurements,^4^ was positioned at depths of 1 and 13cm attached to the RW3 slabs. Here, we used the same setup, with a 0.4mm collimator‐slit, 150MeV protons and 13cm thick RW3‐slabs. Backscatter effects are considered negligible at 1cm in the 13cm setup.^9^, ^14^ Next, we quantitively compared the microDiamond's lateral profile against the lateral profile obtained from the film. We performed a local gamma analysis using 3%/0.2mm and the stricter 3%/0.1mm, criteria recommended for minibeam dosimetry evaluation by Lin et al.,^15^ to account for the high dose changes at submillimetre level. We used a 5% dose cut‐off.

We attached the films to the solid water phantom slabs using adhesive tape, positioned outside the irradiation field.

### 2.3 Alignment and measurement protocol

#### 2.3.1 Alignment

To align the collimator to the beam without misalignments, a four step process was used:

step 1 Perform a coarse initial alignment (to approx. 1mm) of the collimator both in *x*‐direction and *xz*‐plane rotation using the laser alignment guides in the room.^13^
step 2 Rotate the collimator (*xz*‐plane alignment) until the position of the max signal is found.
step 3 Translate the collimator (*x*‐axis alignment) until the position of the max signal is found.
step 4 Measure the lateral profile with the microDiamond to verify no asymmetry is evident in the profile. If asymmetry is present, rotate the collimator slightly until the profile becomes symmetric.

In steps 2 and 3 we visually identified the position of the max signal from the throughput curve using the electron chamber measurement. For the verification in step 4, an absolute symmetry value ≤5% was used as the acceptance criterion.

We re‐aligned the collimator after the initial setup and each time the slit‐width was adjusted, following the recommendations of Ortiz et al.^11^

#### 2.3.2 Measurements

We sampled each point for 1s using the microDiamond to obtain the lateral profile. We performed measurements at 1cm depth, representing the beam entry region, and at 13cm depth, near the Bragg‐peak, representing the target region. The 13cm depth was chosen to avoid measuring in steep dose gradient regions. We used measurement intervals of 75‐100μm at 1cm depth and 400‐500μm at 13cm depth.

To avoid outliers, we discarded and repeated measurements if the Bragg Peak Chamber showed fluctuations exceeding 15%, which indicated large fluctuations in the incident pencil‐beam.

The measurements were converted to relative doses by normalising with the peak dose at 1 and 13cm depth obtained without misalignments, done separately for the 0.4 and 0.7mm slit‐width collimators. Only relative doses are shown, as they are sufficient for the purposes of error analysis and comparison of misalignment effects. Conversion factors for absolute dose measurements are provided in the results for readers who wish to convert relative doses to absolute doses.

#### 2.3.3 Measured misalignments

The misalignments used to obtain profiles of 150MeV protons with 0.4 and 0.7mm slit collimators at 1 and 13cm depths are outlined in Table 2.

**TABLE 2:** Misalignment combinations used to measure output for 150MeV protons (*X*=measured, −=not measured).

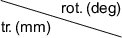	0.0	0.2	−0.2	0.4	−0.4
0.0	X	X	X	X	X
0.5	X	X	X	X	X
1.0	X	X	X	X	X
−1.0	X	—	—	X	X

In addition to 150MeV protons, a reduced set (Table 3) of combinations with the largest misalignments from Table 2 were measured for proton energies of 140, 160, and 170MeV, at 1cm and an adjusted depth to account for the change in position of the Bragg peak. Both 0.4 and 0.7mm slits were used.

**TABLE 3:** Misalignments used to measure output for 140, 160, and 170MeV protons. (*X*=measured, −=not measured).

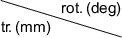	0.0	0.4	−0.4
0.0	X	X	—
1.0	—	X	X

The adjusted depths were calculated based on the $d^{80\%}$ approximation:^16^

(1) $$R\left(E\right)=0.00244E^{1.75},$$

where the range, R, is given in cm, and the proton energy, E, in MeV.

The new phantom depths were then subsequently determined as:

(2) $$d_{\text{phantom,adjusted}}\left(E\right)=\frac{13\;cm}{R(150\;MeV)}\cdot R\left(E\right),$$

which resulted in measurement depths of 11.5, 14.5, and 16.2cm for 140, 160, and 170MeV protons, respectively.

### 2.4 Data processing and analysis

We conducted the data processing in Spyder (v 6.0.5) using Python (v 3.12.7) with numpy (2.1.3), pandas (2.2.3), lmfit (1.3.2), and matplotlib (3.10.3).

#### 2.4.1 Fitting model

Proton minibeams exhibit a different beam‐broadening pattern than conventional pencil‐like proton beams, requiring updated lateral profile parametrisation models.^17^ Savenkov et al.^17^ introduced a new parametrisation model specifically for minibeams by using components used in pencil‐like proton beams models. An overview of several fitting models (including their components) used in pencil‐like proton beams models is given by Bellinzona et al.^18^

We developed a new fitting model specifically tailored to our use case: modeling lateral profiles that include asymmetry resulting from rotational collimator misalignments. Our model is a superposition of:

- A skewed Gaussian to model the primary peak component, which exhibits a skew when the collimator is rotationally misaligned.
- A Gaussian to exclusivel model the asymmetric component resulting from collimator rotations.
- A Cauchy‐Lorentz to model the tail (< 5% peak dose) of the lateral profile.^18^, ^19^

**FIGURE 2:**
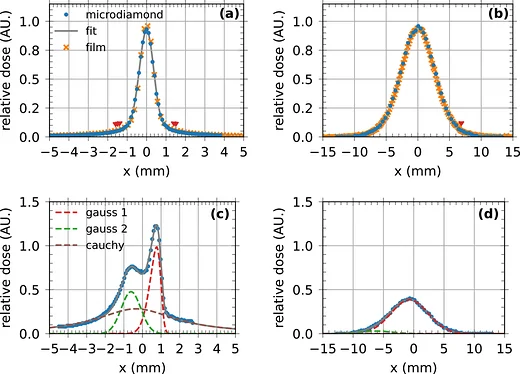
Measurements of the microDiamond shown against film at (a) 1cm and (b) 13cm depth, both acquired using a 0.4mm slit with no translational and rotational misalignment. Points failing the 3%/0.1mm gamma analysis are indicated with a red triangle. No points fail at 3%/0.2mm. (c) and (d) show a microDiamond measurement using the same 0.4mm slit, 1mm lateral translational, and 0.4deg rotation misalignment at 1 and 13cm depth, respectively. Individual fit components are shown in (c) and (d).

An example fit using this model, including the individual fitting components, at 1 and 13cm depth is shown in Figure 2c and 2d, respectively.

To quantify the accuracy of the fits, we used the coefficient of variation (CV)(RMSE):

(3) $$\text{CV(RMSE)}=\frac{\sqrt{\frac{1}{N}\sum_{i=1}^{N}{\left(y_{i}-{\hat{y}}_{i}\right)}^{2}}}{\bar{y}}\times 100\%,$$

where $y_{i}$ is the measured dose, ${\hat{y}}_{i}$ is the fitted dose, N is the number of points measured in the lateral profile, and $\bar{y}$ is the arithmetic mean of the measured dose.

#### 2.4.2 Lateral position correction

Whenever the slit‐width or the phantom slabs were adjusted (i.e., whenever the collimator setup changed or the microDiamond stage was moved), the lateral profile was measured with nominal alignment to align the microDiamond stages with the minibeam central axis such that x=0 always corresponds to the peak dose of the nominally aligned minibeam.

#### 2.4.3 Analysis

From the fitted profiles we determined the maximum dose, $D_{\max}$, the lateral position of the peak dose, $x_{D_{\max}}$, the full width at half maximum (FWHM), and the symmetry. Additionally, we calculated the percentage dose decrease compared to the nominal alignment.

To simulate minibeam arrays with the SSC and multi‐slit collimator (MSC), fitted dose profiles were superposed assuming a parallel incident beam, consistent with the research beamline at HollandPTC.^13^ For the SSC, the profiles were summed across the number of slits, with spatial displacements equal to the CTC distance. The MSC simulation additionally included contributions from neighboring slits irradiated by the incident beam, scaled according to their distance from the central axis. The lateral intensity of the incoming beam was modeled as a Gaussian function, with sigma matched to the research beamline.

With the generated arrays, the minimum number of beams required to achieve a flatness below 3% was determined for a range of CTC distances. We then assessed how misalignments or energy variations affect the compliance with the 95%‐107% criterion following ICRU 78 recommendation for clinical protocols with homogeneous dose prescription.^20^

For the minibeam arrays, the lateral flatness and for the single minibeams and minibeam arrays, the lateral symmetry were calculated according to ICRU 78. Here, flatness was calculated as:

(4) $$\text{flatness}=\frac{D_{\max}-D_{\min}}{D_{\max}+D_{\min}}\cdot 100\%,$$

where $D_{\max}$ and $D_{\min}$ are maximum and minimum dose in the central 80% field width, which is 80% of the FWHM (ICRU 78). Symmetry was calculated as:

(5) $$\text{symmetry}=\frac{\int_{\text{left}}-\int_{\text{right}}}{\int_{\text{total}}}\cdot 100\%,$$

where the integrals represent dose over the left and right regions of the lateral profile. The cut‐off between the left and right area was defined at the location of the (primary) dose peak for a single minibeam and at the center of the central 80% region for a minibeam array, the latter in line with ICRU 78. For flatness, lower values indicate improved flatness, while for symmetry, lower absolute values indicate improved symmetry.

## 3 RESULTS

For our automated setup, measuring a single lateral profile took on average, approximately 60s, with 1s per measurement point and 0.1‐0.5s for stage movement between points. Measurement times varied depending on the spatial sampling interval.

Gamma pass rates were 100% at both 1 and 13cm depths using a 3%/0.2mm criterion. With the much stricter 3%/0.1mm criterion, pass rates were 84% at 1cm and 97% at 13cm. The microDiamond and film measurements, along with the corresponding fits are shown in Figure 2; all points failing the 3%/0.1mm gamma analysis were located in the low‐dose tails.

The overall mean CV of the fitting model was 3.18% (range from 1.14% to 5.64%). CV values per slit‐width, depth, and rotation/no‐rotation are listed in Table 4.

**TABLE 4:** CV(RMSE) values of the microDiamond data fits. Values displayed as: mean (absolute standard deviation).

	0.4 mm slit	0.7 mm slit
	CV (%)	CV (%)
1 cm, no rotation	2.25 (1.19)	4.87 (0.56)
1 cm, rotations	3.58 (0.79)	3.21 (1.14)
13 cm, no rotation	3.62 (0.87)	2.53 (0.25)
13 cm, rotations	3.47 (0.77)	2.35 (0.26)

The conversion factors used to normalize the peak doses to relative doses were 0.273Gy and 0.539Gy at 1cm depth and 0.070Gy and 0.162Gy at 13cm depth for the 0.4 and 0.7mm slit‐width collimators, respectively. The peak doses at the 1cm depth were 3.9 and 3.3 higher than the peak dose at 13cm for the 0.4 and 0.7mm slits, respectively, as expected for minibeams.^6^

The $D_{\max}$, $x_{D_{\max}}$, FWHM and symmetry values for rotational and translational collimator misalignments at 1 and 13cm depth can be can be found in Table 5 (0.4mm slit) and Table 6 (0.7mm slit). Rotational misalignments resulted in large reductions of peak dose and symmetry (i.e., increased the asymmetry), which increases with larger rotations. The asymmetry of a minibeam from rotational misalignments at 1cm depth was larger for the smaller slit (0.4mm) whereas at 13cm depth asymmetry was often larger for the 0.7mm slit. The asymmetry at 1cm depth was much larger than the asymmetry at 13cm depth.

**TABLE 5:** Determined quantities of single minibeam with a 0.4 mm collimator at 1 and 13 cm depth. The peak values are normalised to the nominal alignment at 13 cm depth.

		1 cm					13 cm				
rot. (deg)	tr. (mm)	peak (‐)	peak decr. (%)	peak x‐pos. (mm)	symmetry (%)	FWHM (mm)	peak (‐)	peak decr. (%)	peak x‐pos. (mm)	symmetry (%)	FWHM (mm)
0.0	0.0	3.93	0.0	0.00	3.02	0.88	1.00	0.0	0.00	0.13	6.27
0.0	−1.0	4.03	−2.6	−1.03	0.80	0.88	1.00	−0.4	−1.04	−0.46	6.53
0.0	0.5	4.00	−2.0	0.50	1.10	0.89	0.93	7.3	0.51	−0.03	6.49
0.0	1.0	3.55	9.5	1.03	4.85	0.88	0.93	11.0	0.99	−0.49	6.40
0.2	0.0	2.79	29.0	−0.17	18.13	1.00	0.80	20.4	−0.79	1.30	6.72
0.2	0.5	3.04	22.5	0.30	12.74	0.98	0.79	21.2	−0.18	2.88	6.73
0.2	1.0	2.48	36.8	0.84	15.39	1.03	0.72	27.6	0.31	2.11	6.76
0.4	0.0	1.53	60.7	−0.28	35.59	1.82	0.53	46.7	−1.69	4.01	7.19
0.4	−1.0	1.80	54.2	−1.33	31.14	1.38	0.55	45.3	−2.62	5.06	7.04
0.4	0.5	1.38	64.7	0.24	38.10	1.97	0.46	53.7	−1.22	4.18	7.37
0.4	1.0	1.24	68.4	0.75	37.92	2.07	0.39	61.0	−0.77	3.85	7.43
−0.2	0.0	3.43	12.6	0.22	−8.72	0.94	0.84	16.1	0.72	−1.68	6.74
−0.2	0.5	3.45	12.1	0.73	−7.91	0.92	0.84	16.1	1.24	−1.43	6.63
−0.2	1.0	3.40	13.3	1.25	−6.31	0.91	0.82	17.9	1.74	−1.51	6.58
−0.4	0.0	2.04	48.1	0.32	−29.72	1.16	0.53	47.1	1.62	−3.66	7.19
−0.4	−1.0	1.68	57.1	−0.75	−32.18	1.78	0.50	50.1	0.71	−2.41	7.37
−0.4	0.5	2.09	46.7	0.83	−28.44	1.14	0.54	45.6	2.17	−1.93	7.18
−0.4	1.0	2.13	45.7	1.38	−25.13	1.10	0.55	44.7	2.59	−2.91	7.08

**TABLE 6:** Determined quantities of single minibeam with a 0.7 mm collimator at 1 and 13 cm depth. The peak values are normalised to the nominal alignment at 13 cm depth.

		1 cm					13 cm				
rot. (deg)	tr. (mm)	peak (‐)	peak decr. (%)	peak x‐pos. (mm)	symmetry (%)	FWHM (mm)	peak (‐)	peak decr. (%)	peak x‐pos. (mm)	symmetry (%)	FWHM (mm)
0.0	0.0	3.33	0.0	0.0	0.75	1.08	1.00	0.0	−0.00	0.18	6.44
0.0	−1.0	3.26	2.2	−1.04	0.76	1.11	0.97	2.7	−1.09	0.07	6.46
0.0	0.5	3.32	0.4	0.52	2.01	1.11	0.99	1.2	0.52	0.04	6.48
0.0	1.0	3.22	3.4	1.05	2.85	1.12	0.96	3.7	1.10	0.58	6.46
0.2	0.0	2.74	17.6	−0.11	8.43	1.20	0.88	12.1	−0.41	2.00	6.59
0.2	0.5	2.68	19.5	0.43	11.56	1.20	0.86	14.3	0.11	2.01	6.61
0.2	1.0	2.55	23.5	0.98	13.23	1.21	0.81	18.9	0.69	2.71	6.65
0.4	0.0	2.02	39.5	−0.11	23.61	1.16	0.64	36.0	−0.84	5.50	7.06
0.4	−1.0	2.11	36.6	−1.19	20.87	1.19	0.67	32.9	−1.90	4.99	6.99
0.4	0.5	1.95	41.4	0.4	23.36	1.14	0.61	39.3	−0.29	6.21	7.16
0.4	1.0	1.87	43.8	0.95	24.88	1.11	0.57	42.9	0.24	6.16	7.22
−0.2	0.0	3.07	7.9	0.13	−5.30	1.16	0.95	4.7	0.41	−1.20	6.45
−0.2	0.5	3.13	6.1	0.67	−3.61	1.15	0.96	4.4	0.94	−1.26	6.49
−0.2	1.0	3.13	6.1	1.20	−3.58	1.13	0.95	4.5	1.48	−1.29	6.44
−0.4	0.0	2.29	31.2	0.16	−18.68	1.20	0.75	25.4	0.80	−4.15	6.82
−0.4	−1.0	2.09	37.3	−0.91	−20.47	1.18	0.68	32.0	−0.26	−4.34	6.88
−0.4	0.5	2.35	29.5	0.70	−17.32	1.21	0.76	24.1	1.36	−3.33	6.83
−0.4	1.0	2.42	27.3	1.24	−16.34	1.20	0.78	22.2	1.88	−3.62	6.73

**FIGURE 3:**
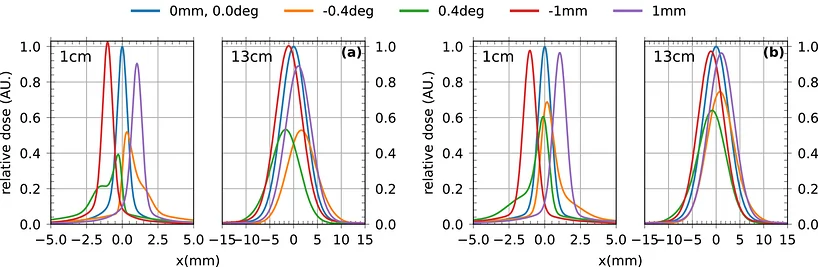
Lateral profiles showing various collimator‐to‐beam misalignments at different depths and slit‐widths: (a) shows a 0.4mm slit width at 1 and 13cm depth; (b) shows a 0.7mm slit width at 1 and 13cm depth.

Lateral profile measurements at 1 and 13cm depth for the nominal alignment, ± 0.4deg rotational and ± 1mm translational misalignments, for both 0.4 and 0.7mm slit widths are shown in Figure 3a and 3b, respectively. At 1cm depth, the rotational misalignments introduce substantial asymmetry and a large dose reduction. As also shown in Tables 5, 6, the dose reductions are more pronounced for the 0.4mm slit compared to the 0.7mm slit. At 13cm depth, the asymmetry from rotational misalignments observed at 1cm has substantially decreased while the dose reductions are still observed. The measurements with translational misalignments show a shift in the profile of similar magnitude as the translation and exhibit only a slight reduction in peak dose at both 1 and 13cm depth.

**FIGURE 4:**
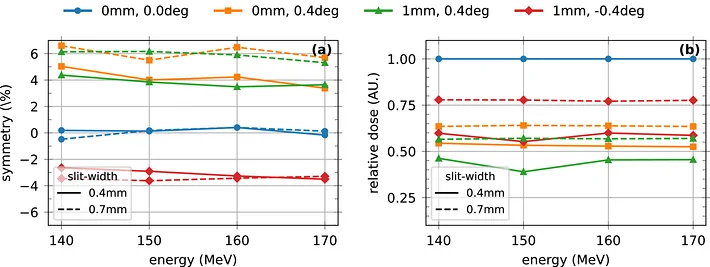
(a) Symmetry and (b) Relative peak dose for 140, 150, 160, and 170MeV protons and 0.4mm (solid lines) and 0.7mm (dashed lines) collimator slit‐widths.

Symmetry and relative peak dose with varying combined translational and rotational collimator misalignments for 140, 150, 160, and 170MeV protons are shown in Figure 4. Variations in symmetry (Figure 4a) and peak dose (Figure 4b) with energy are negligible: misalignments had a greater impact on symmetry and peak dose than energy variations.

**FIGURE 5:**
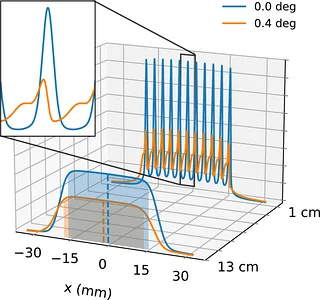
Simulated minibeam array with a SSC, using a 0.4 mm slit, 3.5 mm CTC and 11 beams, for the aligned collimator (blue line) and a rotationally misaligned collimator (orange line). The figure shows the broadening, and flattening, of the arrays at depth. The shaded areas at 13cm denote the central 80% field width.

A simulation of an aligned and misaligned minibeam array with a 0.4mm MSC is depicted in Figure 5. At 1cm depth the dose peaks were lower for the misaligned case while the valley doses were higher. At 13cm depth, the misaligned case shows an overall lower dose and a lateral shift of the entire profile, reducing dosimetric robustness.

**FIGURE 6:**
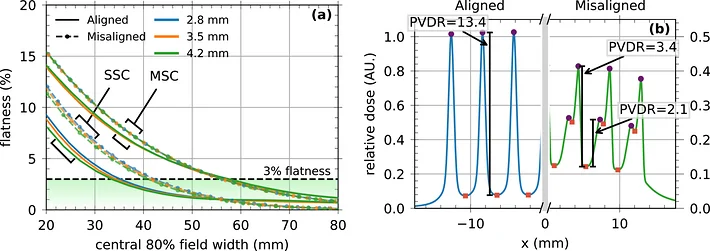
(a) Flatness against central 80% field width for a 0.4mm slit width. Increasing the number of minibeams (larger central 80% field width) improves flatness for both SSCs and MSCs. A minimum field width is required to achieve <3% flatness. The misaligned case represents a 0.4deg rotational and 1mm translational collimator‐to‐beam misalignment. (b) Minibeam array at 1cm depth for a 0.4mm SSC with 150MeV protons. Only three minibeams are shown for each scenario. Left: nominal alignment (blue); right: 0.4deg rotation and 0.5mm translation (green).

Figure 6a shows flatness versus central 80% field width for both the nominal alignment and a misaligned measurement (0.2deg rotation, 1mm translation) for several CTC distances. Increasing the number of minibeams increases the central 80% field width. For both SSCs and MSCs, a minimum field width is required to achieve flatness of 3% for all CTC distances. The crossover, the field width at which flatness falls below 3%, remains nearly constant across different CTC configurations and is smaller for the SSC than for the MSC. For SSC arrays, this crossover point is lower for the aligned cases than for the misaligned cases, whereas for MSC arrays, it is equal for both the aligned and misaligned cases.

Figure 6b shows the measured minibeam array generated with a 0.4mm SSC and 150MeV protons at 1cm depth with a 4.2mm CTC for two scenarios: nominal alignment (left) and an intentionally introduced misalignment of 0.4deg rotation and 0.5mm translation (right). For clarity, only three minibeams are shown for each scenario. The introduced misalignment resulted in additional secondary peaks in the lateral profile, together with reduced primary peak amplitudes and increased valley doses, leading to lower PVDR values (2.1 and 3.4) compared with the nominally aligned case (PVDR 13.4).

## 4 DISCUSSION

In this study we developed a setup to accurately measure the lateral profile of a proton minibeam. The setup, based on the microDiamond detector and automated positioning, substantially reduces measurement time compared to conventional film dosimetry. The setup takes about 10 minutes and can measure a single minibeam lateral profile in under one minute. We validated the setup by comparing measurements to film dosimetry using gamma analysis where we demonstrated a strong agreement between for both the 3%/0.2mm and much stricter 3%/0.1mm criteria. We modeled a single minibeam accurately by superposing two Gaussian components and a Lorentzian (mean CV of 3.18%). Minor local systematic over‐ and underestimations in the residual plots of fits for measurements with rotational collimator misalignments were observed. However, this is expected due to the use of a Gaussian approximation for the secondary peak/asymmetry. Our approach is complementary to existing approaches for managing collimator‐to‐beam alignment. Ortiz et al.^11^ characterized dosimetric effects on misalignments on a clinical pencil‐beam scanning system, while Lin et al.^21^ demonstrated that an integrated snout‐based collimator achieves excellent mechanical stability with rotational reproducibility of 0.1deg on a clinical IBA system. In contrast, our method is detector‐based and system‐independent, and applicable to clinical and research systems that lack integrated collimator positioning. Even on highly stable platforms, residual sub‐degree misalignments cannot be ruled out and, as shown in this study and Ortiz et al.,^11^ can have non‐negligible dosimetric impact for narrow slits. Therefore, a fast and independent verification method remains relevant across different delivery systems. Our methodology can therefore serve as a platform‐independent QA and characterisation tool.

Collimator‐to‐beam misalignments substantially distort the minibeam shape at shallow depths (Figure 3). Translational misalignments mainly result in a lateral shift of the beam profile without changing its shape while rotations lead to large reductions in peak dose and introduce pronounced asymmetry. These effects were more severe for the narrower 0.4mm collimator slit, which showed greater sensitivity to rotational misalignments in terms of asymmetry (at 1cm depth) and peak dose reductions (at both 1 and 13cm depth) compared to the 0.7mm slit. However, at 13cm depth, the 0.7mm slit exhibited more asymmetry than the 0.4mm slit. To explain this, we referred to the findings of Prezado et al.^6^ and Savenkov et al.,^17^ who describe how minibeams undergo lateral scattering and the FWHM becomes “washed out” with depth. Beams with a smaller FWHM undergo more relative lateral scattering with respect to the initial minibeam width, which likely accounts for the stronger asymmetry observed at 13cm for the 0.7mm slit, as it experiences less relative scattering than the 0.4mm slit minibeam. The asymmetry and peak dose reductions, due to rotational and translational misalignments, are also observed for other proton energies (140‐170MeV). Figure 4 shows that the impact of proton energy on the asymmetry and dose reductions is negligible compared to the impact of misalignments. We expect these observations to also hold for other clinical energies. The features, $D_{\max},x_{D_{\max}}$, and asymmetry act as a fingerprint of the underlying misalignment, allowing combined misalignments to be disentangled by reading the translation from the peak displacement and the rotation from the residual asymmetry. Iteratively correcting for the inferred translation and rotation will converge to alignment with the beam.

Rotational misalignments led to a failure in meeting the ICRU 78 dose requirement of 95%‐107% for all cases, due to a substantial dose reduction. This indicates the need to mitigate misalignments for clinically acceptable dose coverage at the target. Since the asymmetry due to rotational misalignments reduces over depth, we found that rotational misalignments had a minimal impact on the homogeneity of the dose at larger depths when the central 80% of the nominal alignment, for the same CTC and number of beams, also had a flatness below 3%. In that case, only a lateral shift and dose reduction were observed across the central 80% width, as can be seen in Figure 5. Since homogeneous dose deposition at depth remains achievable in most cases, we propose two possible mitigations strategies for clinical implementation: either align according to the treatment plan; or adapt the plan to possible misalignments. However, this second approach may reduce some benefits of the spatial dose modulation inherent to minibeams, due to the asymmetry, reduced peak doses, and increased valley doses. In both our SSC and MSC arrays, we observed that for a given collimator position (nominal or misaligned), the central 80% field required to achieve a flatness below 3% remained nearly constant across different CTC distances (Figure 6a). This suggests there is a minimum field width to achieve homogeneous dose depositions, implying that clinical targets cannot be too small for a given slit‐width. This field width was larger for MSCs compared to SSCs

We observed that rotations can introduce lower secondary peaks in the lateral profile at shallow depth (1cm), illustrated in Figure 6b. This impacts greatly the defining or reporting of valleys, peaks, and PVDR for evaluation of pMBRT treatment plans, as plans can have greatly different values depending on whether misalignments and hence such secondary peaks are present. Currently there are no guidelines in the literature on how to define valleys, peaks, and, PVDR appropriately, which is important for cases with secondary peaks or highly varying peak/valley ratios. As these parameters are most often reported as a single mean value, this raises the question on how to interpret them in clinical settings and how to relate these values to clinical outcomes. There are ongoing debate in the field regarding the optimal dosimetric parameters, such as Fernandez‐Palomo et al.^22^ and Garcia et al.^23^ who suggest that valley dose may be more predictive of therapeutic benefit, in terms long‐term survival.

Our findings also directly translate to heterogeneous minibeam configurations at the target. In particular, rotation‐induced dose reductions and resulting lateral profile asymmetries extend across the full transmission field and may alter the therapeutic performance of transmission beams. Furthermore, the reduction in PVDR with rotational misalignments suggests that the profile might become more homogeneous at shallower depths, which could be especially critical for beams that require highly heterogeneous beams. Strategies such as interleaving^24^ and scissoring,^25^ which are used to improve target coverage and conformity, are more susceptible to misalignments. This is attributed not only to imperfect overlap of the minibeams but also to the severe dose degradation as observed in our study. Recent collimator designs, such as the dynamic collimators of Sotiropoulos et al.^26^ and Reaz et al.^27^ utilising the Moiré effect, are more complex and can become more sensitive to misalignments. The effects of collimator misalignments are expected to compound when using fractionation, where varying misalignments may occur between treatment fractions. Collimator misalignments must be accounted for in clinical practice.

Our study focussed on the behavior of a single slit with a SSC and a parallel MSC. More complex configurations, for example divergent MSCs or dynamically modulated beams, were beyond the scope and were not evaluated here. Previous research has shown that the orientation of slits within the collimator can substantially affect the minibeam array and its sensitivity to misalignments. Notably, Reaz et al.^12^ demonstrated that rotational misalignments disrupt homogeneous dose deposition at depth for divergent MSCs. In contrast, our findings show that homogeneity is maintained at depth for parallel MSCs. The impact of collimator misalignments may also depend on the angular distribution of the beam. Our simulations assumed a parallel incident beam, so further investigation is needed to confirm whether our findings apply to divergent beams. Our measurements used a single static pencil beam, whereas clinical delivery typically uses a scanned field where averaging over multiple spot positions can occur. This is expected to partially mitigate local asymmetries. Further investigation is warranted to quantify the impact of misalignments under scanned delivery. Thus, the specific collimator geometry, in combination with the beams angular distribution, plays a pivotal role in shaping the minibeam and understanding the behavior of individual slits can provide insights into the behavior of the overall slit configuration.

Positive and negative translational and/or rotational misalignments of equal magnitude did not always produce identical lateral profiles (Tables 5, 6). This can be attributed to inherent beamline asymmetries, whereby the beam shape deviates from an ideal Gaussian profile, and small residual detector positioning uncertainties that remain even after applying our precise alignment procedure. Consequently, the system is not perfectly symmetric. This should be considered when interpreting the reported peak dose reductions and asymmetric metrics for misaligned configurations.

Profiles were acquired using a fixed sampling time per point, with corrections applied for beam fluctuations using the Bragg Peak Chamber, rather than at fixed monitor units (MU). This favors acquisition speed and is sufficient for the relative misalignment analysis reported here, while fixed‐MU delivery remains preferable for absolute dosimetry.

We investigated the effect of rotations (*xz*‐plane) of the collimator. Rotational misalignments in the other two directions also influence the beam output, although their impact is expected to be smaller. The effects of *xy*‐plane rotations are expected to increase with distance away from the central axis (i.e., with increasing field size) because of a larger displacement under the same angle. Rotations in the *yz*‐plane are expected to have a much smaller effect on beam output than *xz*‐ and *xy*‐plane rotations because the slit opening is not rotated with respect to the incoming beam for slit‐based collimators.

In our study, we did not focus on detailed LET effects, particularly in regions with high LET gradients, such as near the distal Bragg peak region, and this represents a limitation of this study. The measurements in this work were performed approximately 1cm before the Bragg peak, where LET variations are less pronounced compared with the distal region. As the analysis focussed on relative output changes under consistent beam conditions, these effects are not expected to alter the primary conclusions. Nevertheless, we recognize that LET effects may still influence the dose distribution at these depths, and this warrants further investigation in future work that aims to assess more comprehensive dose effects in the Bragg peak region.

Despite these limitations, our approach provides important insights into the impact of collimator misalignments on pMBRT delivery and how we can detect such changes in the lateral profile. Our results provide more insights to establish a clinical collimator setup at HollandPTC. Moreover, our methodology is quick to set up and can measure lateral profile quickly, especially compared to the gold standard of film dosimetry, which typically takes 24h to develop. This supports translation into clinical workflows before treatments. Further research is needed to reproducibly map the profiles to underlying misalignments. Our approach showed that the extent of asymmetry in the lateral profile indicates the extent of rotational misalignment. Full quantification and automation are left for future work, to which the system developed here is well suited. The automated nature of our setup also enables the measurement procedure to be easily repeated and adapted, minimising the need for manual adjustments for each measurement, hence reducing radiation exposure for the technicians.

## 5 CONCLUSION

Lateral dose profiles of pMBRT are substantially affected by collimator‐to‐beam misalignments, especially rotational misalignments, in agreement with prior dosimetric studies. Rotational misalignments result in high levels of asymmetry at shallow depths, which reduces over depth due to beam‐widening, transforming into a lateral shift of the profile. For homogeneous dose deposition, the impact on dose homogeneity at depth depends on the specific geometry of both the beam and the collimator, whereas for heterogeneous dose deposition, the heterogeneity is affected along the entire beam path.

Our findings of a single minibeam can be extended to other slit‐based collimators and beam source directions by considering the contribution and effects at each individual slit.

We developed a simple measurement setup that can identify and approximate the magnitude of translational and rotational misalignments in under one minute using commercially available detectors. This approach is well suited for integration into routine clinical QA workflows.

## CONFLICT OF INTEREST STATEMENT

The authors declare no conflicts of interest.
